# No1LikesU! – A Pilot Study on an Ecologically Valid and Highly Standardised Experimental Paradigm to Investigate Social Rejection Expectations and Their Modification

**DOI:** 10.32872/cpe.v2i2.2997

**Published:** 2020-06-30

**Authors:** Lisa D’Astolfo, Lukas Kirchner, Winfried Rief

**Affiliations:** a Department of Clinical Psychology and Psychotherapy, Philipps-University of Marburg, Marburg, Germany; Philipps-University of Marburg, Marburg, Germany

**Keywords:** ViolEx-Model, expectation violation, expectation persistence, expectation modification, dysfunctional expectations, social rejection, No1LikesU!

## Abstract

**Background:**

Dysfunctional expectations have been suggested as core features in the development and maintenance of mental disorders. Thus, preventing development and promoting modification of dysfunctional expectations through intervention might improve clinical treatment. While there are well-established experimental procedures to investigate the acquisition and modification of dysfunctional performance expectations in major depression, paradigms for investigating other important types of dysfunctional expectations (e.g. social rejection expectations) are currently lacking. We introduce an innovative associative learning paradigm, which can be used to investigate the development, maintenance, and modification of social rejection expectations.

**Method:**

A pilot sample of 28 healthy participants experienced manipulated social feedback after answering personal questions in supposed webcam conferences. While participants repeatedly received social rejection feedback in a first phase, differential feedback was given in a second phase (social rejection vs. social appreciation). In a third phase, explicit social feedback was omitted.

**Results:**

Participants developed social rejection expectations in the first phase. For the second phase, we found an interaction effect of experimental condition; i.e. participants adjusted their expectations according to the differential social feedback. In the third phase, learned social expectations remained stable in accordance to the social feedback in the second phase.

**Conclusion:**

Results indicate that the paradigm can be used to investigate the development, maintenance, and modification of social rejection expectations in healthy participants. This offers broad applications to explore the differential acquisition and modification of social rejection expectations in healthy vs. clinical samples. Further, the paradigm might be used to investigate therapeutic strategies to facilitate expectation change.

## Abstract (highlights)

- This paradigm can be used to induce and modify social rejection expectations.
- This allows to investigate differences in expectation acquisition, maintenance, and modification between clinical vs. healthy samples.
- Further, this paradigm enables research on interventions promoting expectation modification.

Recent developments in clinical psychology propose dysfunctional expectations (i.e. future-directed ‘if-X-then-Y’-predictions, Rief et al., 2015, p. 380) as an important factor in the development of mental disorders and as a promising target in clinical treatment (e.g. Greenberg, Constantino, & Bruce, 2006; Rief & Glombiewski, 2017; Rief et al., 2015).

Dysfunctional expectations have been shown to play a crucial role in mental health as they negatively impact future behaviour (e.g. excessive avoidance, Krypotos, 2015), aggravate subjective suffering (e.g. pain perception, Jepma, Koban, van Doorn, Jones, & Wager, 2018), and elicit potentially maladaptive anticipatory reactions (e.g. negative mood, Davidson, Marshall, Tomarken, & Henriques, 2000).

Further, dysfunctional expectations have been shown to impede important clinical outcomes (e.g. treatment success, Constantino, Vîslă, Coyne, & Boswell, 2018). As George A. Kelly put it early in his theory of personal constructs: ‘A person’s processes are psychologically channelised by the ways in which he anticipates events’ (Kelly, 1977, pp. 358-359). Thus, preventing acquisition and promoting modification of dysfunctional expectations through intervention might improve clinical treatment (Craske, Treanor, Conway, Zbozinek, & Vervliet, 2014; Rief & Glombiewski, 2016; Rief & Joormann, 2019).

However, acquisition, maintenance, and modification of dysfunctional expectations is still little understood (Rief & Joormann, 2019). While there are promising theoretical approaches (Kube, Rief, & Glombiewski, 2017; Kube, Schwarting, Rozenkrantz, Glombiewski, & Rief, 2020) and well established experimental procedures concerning this issue with regard to dysfunctional performance expectations in major depression (Kube, Rief, Gollwitzer, & Glombiewski, 2018), experimental paradigms are lacking when it comes to other types of dysfunctional expectations (see Liebke et al., 2018, for a laudable exception).

Since especially (dysfunctional) expectations of social rejection (e.g. ‘When I open myself to others, they will refuse me!”) have serious implications for mental health (e.g. Bianchi, Schonfeld, & Laurent, 2015; Gao, Assink, Cipriani, & Lin, 2017) and the course of various mental disorders (e.g. Bungert et al., 2015; De Panfilis, Riva, Preti, Cabrino, & Marchesi, 2015; Kimbrel, 2008; Slavich, O’Donovan, Epel, & Kemeny, 2010), ecologically valid experimental procedures are strongly needed for further investigation.

The aim of the current study was to develop an experimental social rejection expectation paradigm (No1LikesU!), which can be used to investigate the acquisition, maintenance and modification of social rejection expectations within a highly standardised and ecologically valid procedure. In contrast to existing paradigms on social exclusion (for an overview, see Riva & Eck, 2016), No1LikesU! was especially designed to mimic key processes proposed by a recently published theoretical model on expectation development, maintenance, and modification – the so called ‘ViolEx-Model’ by Rief and colleagues (2015).

This model proposes that when entering concrete situations, individuals form situation-specific predictions about these situations (drawn from more generalised expectations) which become either (a) confirmed or (b) disconfirmed by experience. While repeated expectation confirmations should stabilise or reinforce the original situation-specific prediction (or respectively, the underlying generalised expectation), repeated expectation ‘violations’ should entail its modification (Gollwitzer, Thorwart, & Meissner, 2018; Rief et al., 2015).

Following the predications of the model, we hypothesise that (1) repeatedly exposing healthy individuals to situation-specific experiences of social rejection will increase levels of social rejection expectations over time. Consistent with the ViolEx-Model, we further hypothesise that (2) repeatedly exposing healthy individuals with increased levels of social rejection expectations to situation-specific experiences of social rejection (‘Stabilisation’) vs. appreciation (‘Modification’) will lead to differential changes (i.e. to an increase vs. stabilisation) in social rejection expectation levels over time.

## Method

No1LikesU! is an ecologically valid and highly standardised associative learning paradigm created to model the development, maintenance, and modification of social rejection expectations. Like the O-Cam paradigm (Godwin et al., 2014; Goodacre & Zadro, 2010), it relies on a cover story leading participants to believe that they are going to interact with real human beings via webcam. Participants in No1LikesU! are told that they are going to participate in a study investigating ‘how people socialise with and affect each other in virtual environments’. Participants pass multiple supposed ‘webcam-conferences’ (actually, realistic looking video stimuli) on a computer in which they answer personal questions to different ‘listeners’ (actually, pre-recorded and instructed confederates). Afterwards, they receive written social feedback on their self-presentation (actually, manipulated feedback that induces experiences of social rejection vs. social appreciation). 

The local ethics committee (reference number 2018-36k) approved the study. All participants gave written informed consent before they started the experiment. This study was part of a parent study, which additionally investigates interventions for promoting the modification of dysfunctional expectations. In the present work, we focus on the effects of the paradigm on the development, maintenance, and modification of social rejection expectations in healthy participants.

### Participants

We recruited participants via e-mail lists, flyers, and the research participation system of our university. Inclusion criteria were: (a) A minimum age of 18 years, (b) sufficient German language skills, (c) no severe visual impairment, (d) no serious physical illness, (e) no current psychological stress, (f) not in psychotherapeutic treatment, and (g) a sum score in Beck’s Depression Inventory II (BDI-II; Kühner, Bürger, Keller, & Hautzinger, 2007) ≤ 13, indicating no to minimal depressive symptoms.

Until now, a pilot sample of 31 healthy participants could be included in the study, which provides sufficient power to investigate our hypotheses (Huta, 2014). As mentioned above, recruitment based on a priori power analyses continues as we test No1likesU! within an ongoing study addressing further research questions we do not fully report here (preregistered at ‘Aspredicted’: https://aspredicted.org/g544c.pdf). Since recruitment is currently faltering for the parent study, we would like to publish our pilot results on the paradigm contrary to preregistration in order to make them accessible to the research community. Three participants had to be excluded due to technical problems with the experimental software. The final pilot dataset consisted of 28 healthy participants (82.10% female, *M*_age_ = 23.39 years, *SD* = 6.51, range of age = 19–51 years). Table 1 shows the demographic data of the sample.

Participants received credit points as compensation for their participation. Alternatively, they got the chance to win gift vouchers for different online shops.

### Procedure

**Figure 1:**
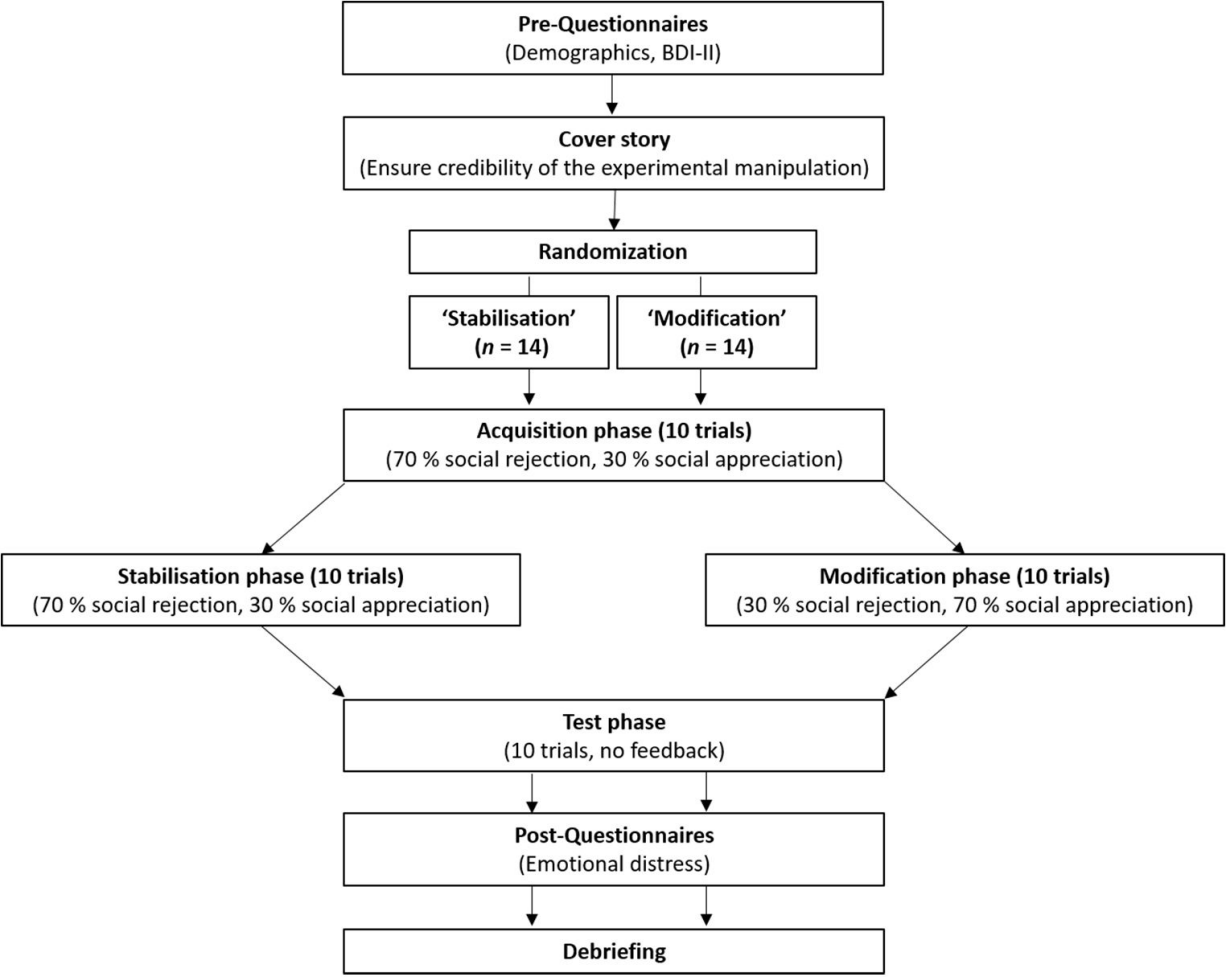
Study Design

Testing sessions started with participants reading the study information and signing informed consent (see Figure 1 for an overview of the study design). Afterwards, they completed paper-pencil pre-questionnaires.

Research assistants checked age as well as BDI-II cut-off scores. Participants who failed the inclusion criteria received partial compensation and were fully debriefed. Participants who met the inclusion criteria received study information incorporating the cover story. To allay concerns about the authenticity of the webcam conference, participants were told that their listeners (who were announced as ‘students from an experimental internship at the university’) were instructed ‘not to talk’ during conferences for ‘methodological reasons’. Afterwards, research assistants started the paradigm on the computer and left the experimental room. The participants were fully randomised into two independent experimental conditions (group ‘Stabilisation’ vs. group ‘Modification’) and followed instructions presented on the computer screen, which guided through the paradigm.

To model key processes of the ‘ViolEx-Model’, No1LikesU! encompasses multiple trials (30) which are divided into three different experimental phases (acquisition phase, stabilisation vs. modification phase, test phase, see Figure 1).

These phases are structurally based on fear conditioning paradigms (Lissek et al., 2005; Lonsdorf et al., 2017). During the acquisition and stabilisation phase, participants repeatedly form associations between introducing themselves to strangers (conditioned stimulus, CS) and being socially rejected (unconditioned stimulus, US) resulting into situation-specific social rejection expectations (conditioned response, CR). During modification, opposing associations (CS-US’ [being socially appreciated]) are formed resulting into expectations of social appreciation. In order to enhance stability of expectations and ecological validity, No1LikesU! provides partial reinforcement (70%) within these phases. To explore the stability of the social expectations learned within the experimental paradigm, No1LikesU! ends with a test phase which did not provide written social feedback (retention test).

After completing the paradigm, research assistants entered the experimental room and provided paper-pencil post-questionnaires to check for suspiciousness about the cover story and emotional distress due to participation. Participants were then fully debriefed about the true purposes of the study and the deceptions within the experimental manipulation. Testing sessions lasted between 1.0 and 1.5 hours.

### Measures

Note that we applied additional questionnaires to address further research questions in the parent study, which we do not describe here.

#### Situation-Specific Social Expectations

We assed situation-specific social expectations using a one-item 7-point bipolar Likert scale (social expectation rating: ‘Please indicate to what extent you expect your next listener to be interested or disinterested in you!’) ranging from -3 (maximal disinterest) to +3 (maximal interest) before each trial. Thus, lower values indicate higher social rejection expectations.

#### Situation-Specific Social Experience

To examine how participants actually perceived a passed webcam conference, we used a one-item 7-point bipolar Likert scale (social experience rating: ‘Please indicate to what extent you experienced interest or disinterest from your last listener!’) ranging from -3 (maximal disinterest) to +3 (maximal interest) after each trial. Thus, lower values indicate higher social rejection experiences.

#### Pre-Questionnaires

##### Depressive Symptoms

We assessed depressive symptoms using the Beck Depression Inventory II (BDI-II; Kühner et al., 2007 prior to running No1LikesU!). Participants responded to the 21 items on a 4-point scale ranging from 0 to 3. The sum score of the 21 items ranges between 0 and 63, whereby higher values indicate more depressive symptoms.

##### Socio-Demographics

We used a brief self-report questionnaire in order to assess demographic variables like sex, age, nationality, relationship status, educational level, employment status, and living situation.

#### Post-Questionnaires

##### Emotional Distress Due to Participation

We assessed emotional distress due to participation by asking whether participants felt impaired due to the experimental procedures (‘Do you feel impaired due to our investigation?’). Further, we applied a one-item 5-point bipolar Likert scale (‘Please indicate to what extent you feel positive or negative in this moment!’) ranging from -2 (very negative) to +2 (very positive) to assess emotional distress. Higher values indicate lower emotional distress due to participation.

##### Suspiciousness

In order to assess the credibility of the cover story, the video stimuli and the experimental manipulation, we asked participants whether 1) they knew any of their ‘webcam partners’, 2) what they believed was the aim and purpose of the study, and 3) how they experienced the experimental procedure. Responses were rated on a 3-point Likert scale ranging from 0 ("not suspicious at all") to 2 ("doubted the authenticity of the webcam conferences").

### Apparatus and Stimuli

**Figure 2:**
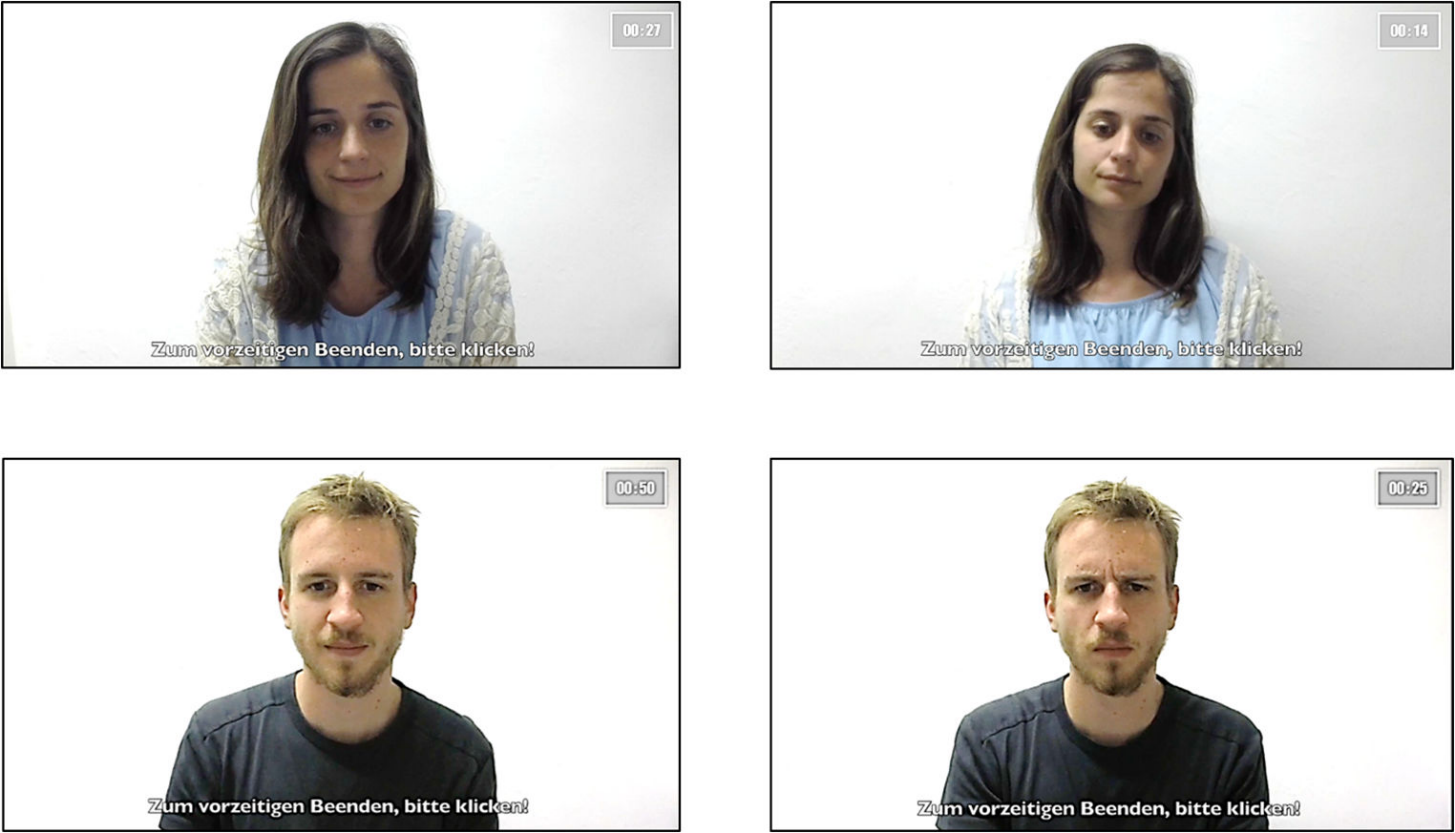
Video Stimuli (Left: Social Appreciation, Right: Social Rejection)

Participants were seated in front of a computer with an external microphone and a webcam connected to the computer. The paradigm, including instructions, video stimuli, and social feedback, was presented on the computer screen. Participants used a mouse to interact with the computer. Video stimuli were pre-recorded with 30 volunteers (15 male, 15 female, age: 25 – 35). Volunteers were instructed to express nonverbal cues of either social rejection or social appreciation (see Figure 2). We produced two sequences of each volunteer resulting into 30 sequences of social rejection and 30 sequences of social appreciation (50 seconds each). The nonverbal feedback during each trial was matched with the written feedback. The personal and self-related questions were adapted from various dating websites in order to promote positive self-disclosure (see Appendix A in the Supplementary Materials). The video stimuli as well as the personal questions were presented fully randomised during experimental procedure in accordance with the partial reinforcement. For each participant, video stimuli and personal questions were never repeated twice.

#### Trial Sequence

**Figure 3:**
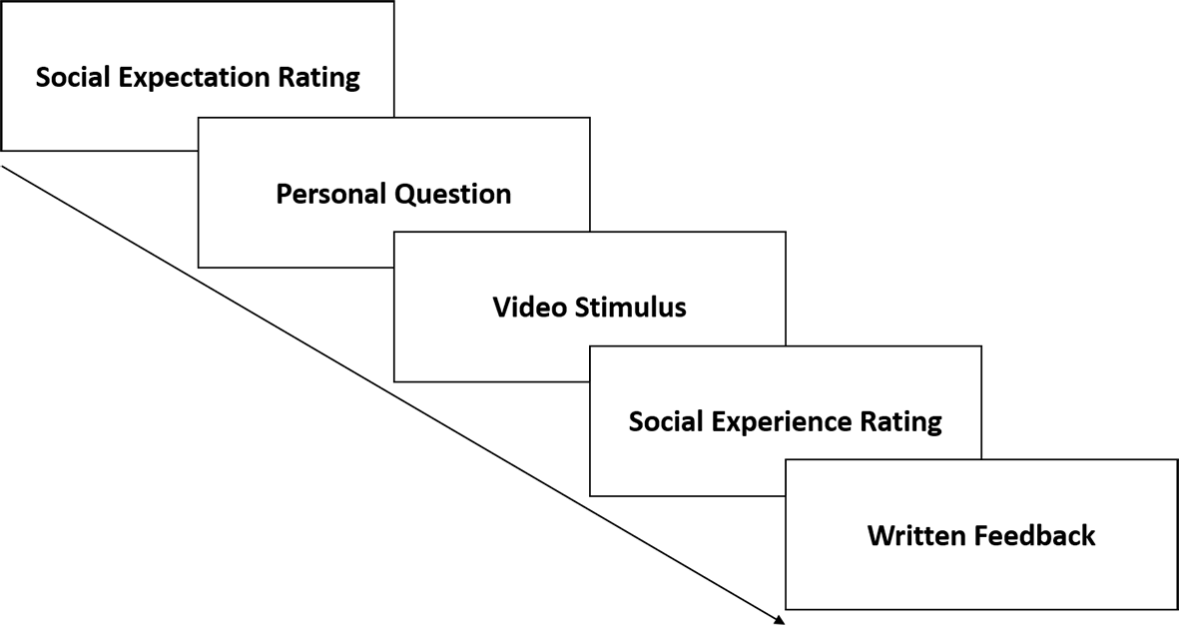
Trial Sequence

Figure 3 gives an overview of the trial sequence. Each trial started with a situation-specific social expectation rating. Afterwards, participants received a personal, self-related question (e.g. ‘What are your hobbies?’) on the screen ostensibly asked by the ‘next listener’ in order to pre-set the content of the next conference. Following preparation time depicted by a countdown (20 seconds), participants received a short connection-signal on the screen (5 seconds) before the supposed ‘webcam conference’ started by showing a pre-recorded video stimulus. To ensure the authenticity of the conferences, participants were instructed to actively end conferences when they finished their self-presentation. After each conference, participants gave a situation-specific social experience rating before receiving written social feedback (e.g. ‘Your last listener found you rather uninteresting and would not like to get in touch with you again.’). This trial sequence was repeated (10 times) within each of the three experimental phases. However, written feedback was omitted in the last phase.

### Statistical Analyses

Before conducting the analyses, we checked for outliers to exclude influential data points. For each expectation rating, we calculated the Mahalanobis distance which we checked against a χ^2^-cut-off of α = .001. We found no influential data points.

All analyses were computed using *R Studio* (R Studio Team, 2015) for R (R Development Core Team, 2008). We used *lme4* (Bates, Mächler, Bolker, & Walker, 2015), *nlme* (Kuznetsova, Brockhoff, & Christensen, 2017), *blme* (Chung, Rabe-Hesketh, Dorie, Gelman, & Liu, 2013), and *lmerTest* (Kuznetsova et al., 2017) to perform a hierarchical mixed effects analysis of the relationship between social expectations, measuring time and experimental condition. Since the times at which expectations were measured are separable into the three phases, we defined a contrast matrix for Time, which accounted for the nested data structure. We used the contrast matrix for Time as a Level-1-fixed effect, and group as a Level-3-fixed effect (including the interaction term). As random effect, we implemented intercepts for participants (Level 2). We checked homoscedasticity and normality via the residual plots, which always showed expected patterns. We obtained *p*-values by likelihood ratio tests, testing the model with the additional level effect against the model without the additional level effect.

Subsequently, we analysed the phases individually to estimate effect sizes for each phase effect. We used linear models to investigate the relationship between social expectations and group affiliation. We entered Group as fixed effect. We inspected the residual plot to check homoscedasticity and normality. Again, all plots showed patterns as expected. For all analyses, we applied sum contrasts to calculate intercepts and slopes.

## Results

### Sample Characteristics

Participants were predominantly young (*M*_age_ = 23.39, *SD =* 6.51), female (82.14%) and well-educated (100% general qualification for university entrance). The mean BDI-II sum score was 4.54 (*SD =* 3.26), indicating that no participant exceeded the clinical threshold of depressive symptoms (Kühner et al., 2007). Table 1 gives an overview of the sample characteristics. There were no significant differences between the experimental conditions in any of the assessed variables.

**Table 1:** Sample Characteristics

Variable	Stabilisation (*n* = 14)	Modification (*n* = 14)	Difference between experimental conditions
Age in years, *M (SD)*	21.79 (3.02)	25.00 (8.57)	*t* (26) = 1.32, *p* = .20
Sex, *N* (%)			χ^2^ = 2.19, *p* = .14
Male	4 (28.57)	1 (7.14)	
Female	10 (71.43)	13 (92.86)	
Nationality, *N* (%)			χ^2^ = 0.37, *p* = .54
German	13 (92.86)	12 (85.71)	
Other	1 (7.14)	2 (14.29)	
Romantic relationship, *N* (%)			χ^2^ = 1.29, *p* = .26
Yes	5 (35.71)	8 (57.14)	
No	9 (64.29)	6 (42.86)	
Living situation, *N* (%)^a^			χ^2^ = 0.01, *p =* .94
Living alone	2 (14.29)	2 (15.38)	
Living with others	12 (85.71)	11 (84.62)	
Educational level, *N* (%)			χ^2^ = 1.71, *p* = .19
University degree	2 (14.29)	5 (35.71)	
No university degree	12 (85.71)	9 (64.29)	
Employment status, *N* (%)			χ^2^ = 1.47, *p* = .23
Employed	6 (42.86)	3 (21.43)	
Not employed	8 (57.14)	11 (78.57)	
BDI-II sum-score, *M (SD)*	4.86 (3.44)	4.21 (3.17)	*t* (26) = - 0.52, *p* = .61
MSER before first trial, *M (SD)*	3.86 (1.29)	4.21 (1.12	*t* (26) = 0.78, *p* = .44
Emotional distress after participation, *M (SD)*	3.21 (0.70)	3.57 (0.65)	*t* (26) = 1.40, *p* = .17

*Note.* BDI-II = Beck Depression Inventory II; MSER = Mean social expectation rating.

^a^One missing data point.

### Manipulation Check for the Nonverbal Social Feedback

We investigated whether the nonverbal social feedback (rejection vs. appreciation) displayed in the videos affected the situation-specific social experience ratings of the supposed webcam conferences. Participants provided these ratings after each conference and before receiving written social feedback.

First, we performed a mixed ANOVA using a linear model of the mean social experience ratings as a function of Group (between factor) and Time (within factor) using Greenhouse-Geisser correction. We found a significant interaction of Group and Time (*F*(1, 33) = 5.09, *p* = .023) as well as a significant main effect for Group (*F*(1, 26) = 4.68, *p* = .040), and Time (*F*(1, 33) = 6.80, *p* = .009).

Next, we performed post-hoc analyses and pairwise comparisons to further analyse the significant interaction effect.

The Bonferroni adjusted *p*-values suggest that the main effect of Group was significant during modification vs. stabilisation phase (*F*(1, 26) = 11.33, *p* = .006) but not during acquisition phase (*F*(1, 26) = 2.46, *p* = .387), and test phase (*F*(1, 26) = 0.64, *p* = 1.000). Pairwise comparisons showed that the mean social experience rating between group ‘Stabilisation’ and group ‘Modification’ differed only during modification vs. stabilisation phase (*p* = .002) when differential nonverbal social feedback was applied (70% social rejection feedback in group ‘Stabilisation’ vs. 70% social appreciation feedback in group ‘Modification’). As expected, group ‘Modification’ (*M* = 3.53, *SD* = 0.64) showed higher social experience ratings than group ‘Stabilisation’ (*M* = 2.77, *SD* = 0.55), indicating more perceived social appreciation.

Regarding the main effect of Time, the Bonferroni adjusted *p*-values suggested significant differences for group ‘Modification’(*F*(1, 16) = 8.26, *p* = .014), but not for group ‘Stabilisation’ (*F*(1,16) = 4.56, *p* = .080). Pairwise comparisons revealed differences in mean social experience rating within group ‘Modification’ between Acquisition Phase (*M* = 3.07, *SD* = 0.72) and Modification vs. Stabilisation Phase (*M* = 3.53, *SD* = 0.64) as well as between modification vs. stabilisation phase and Test Phase (*M* = 3.27, *SD* = 0.58) with modification phase having the highest social experience ratings reflecting the highest nonverbal social appreciation feedback of 70%. We found no significant differences in social experience ratings between acquisition and test phase. These results indicate that the participants experienced the nonverbal social feedback as intended.

### Main Analyses

**Figure 4:**
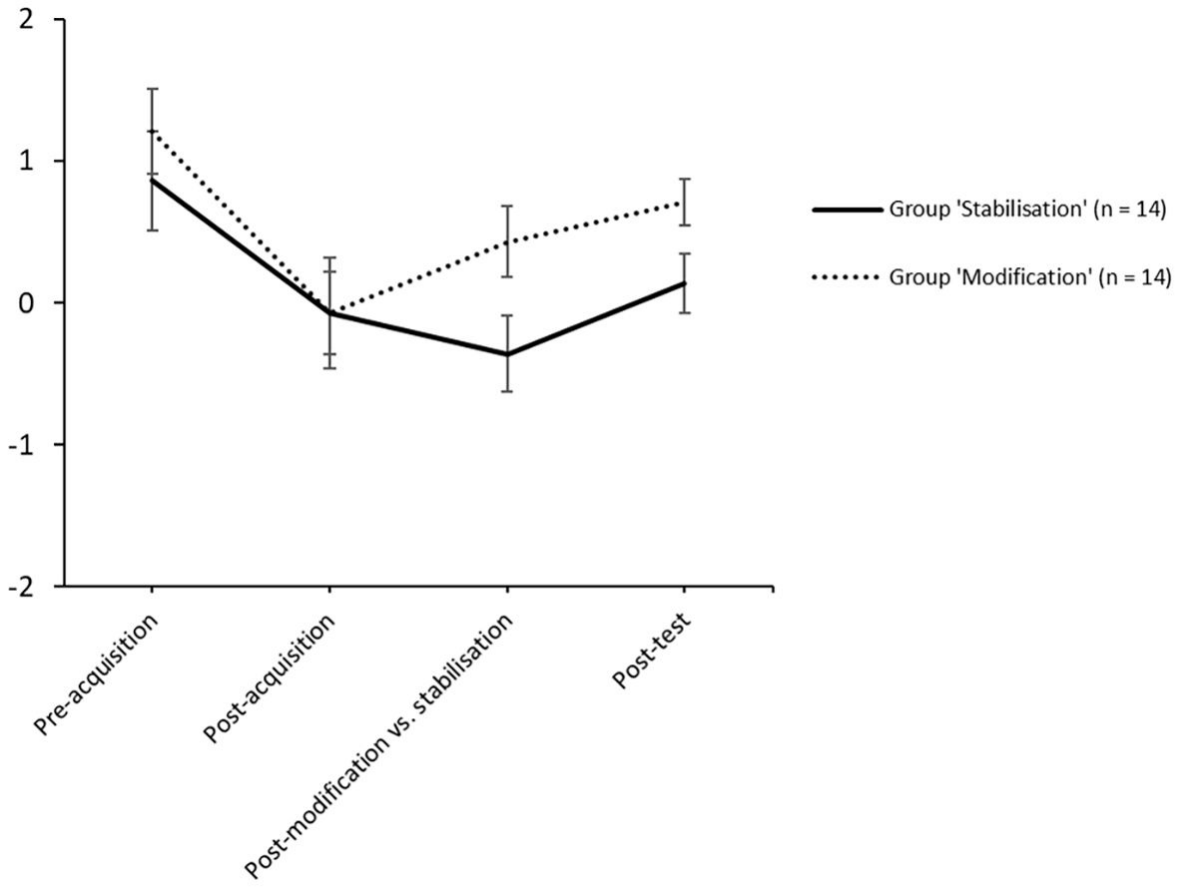
Mean Social Expectation Rating Across All Experimental Phases as a Function of Experimental Condition *Note.* Error bars indicate ± 1 *SE*.

First, we included all experimental phases in one statistical model and investigated changes in social expectation ratings across the course of the experiment. Therefore, we performed a multilevel mixed effect multinomial linear regression on the social expectation ratings as a function of Group and Time (i.e. the contrast matrix of individual social expectation ratings nested in each phase). Time therefore consists of three variables each representing an experimental phase (acquisition phase, modification vs. stabilisation phase, test phase). Unless otherwise stated, we used the standard bound optimisation by quadratic approximation (BOBYQA) optimisation for the models. We calculated the linear regression of the social expectation ratings as a function of Time (Level 1). We then subsequently added the next-level effects until arriving at the full model including Time (Level 1), random intercept for participant (Level 2), and Group with interaction term for Time (Level 3). We compared mixed-effects models using likelihood ratio tests. Here, we will describe the results of the Level-3-model, the results for the Level-1- and Level-2-models can be found in the Supplementary Material. Figure 4 shows the course of the mean social expectation ratings across all phases of the experiment.

The Level-3-model revealed no significant Group x Acquisition Phase interaction (β = -.00, *t* = -0.05, *p* = .585) but a trend for the Group x Test Phase interaction (β = -.02, *t* = -1.96, *p =* .050) as well as a significant interaction for Group x Modification vs. Stabilisation Phase (β = .02, *t* = 2.12, *p =* .034) in accordance with our hypotheses. Also, we found a main effect for Group (β = .15, *t* = 2.00, *p =* .046), Acquisition Phase (β = -.06, *t* = -3.61, *p* < .001), and Test Phase (β = .04, *t* = 2.40, *p* = .016), but not for Modification vs. Stabilisation Phase (β = -.00, *t* = -0.5, *p* = .572). In other words, there were no significant group differences in social expectation ratings during acquisition phase but during stabilisation vs. modification phase and test phase (retention test), whereby participants in group ‘Stabilisation’ showed higher social rejection ratings than participants in group ‘Modification’. Also, social rejection ratings significantly increased during acquisition phase and slightly decreased during test phase for both groups. The non-significant main effect for Stabilisation vs. Modification Phase can be explained with the opposing effect of the groups on social expectation ratings due to the inverted reinforcement rates.

Table 2 shows the model comparisons for the hierarchical linear regression. The models were sequentially tested against the previous models.

**Table 2:** Analysis of Variance for the Hierarchical Linear Regression Models

Model	AIC	χ^2^	χ*_df_*	*p*
Level 2 (random effect for participant)	2249.7	–	–	–
Level 3 (fixed effect for group)	2243.8	13.87	4	.007

*Note.* AIC = Akaike information criterion.

### Individual Phases

Next, we used MANOVA tests to investigate the effect of Group on the social expectation ratings for each phase individually to investigate the effect sizes of the changes.

#### Hypothesis 1: Main Effect of Acquisition Phase

We constructed a linear model of the social expectation ratings (as outcome matrix for ratings 1 to 10) as a function of Group and Baseline Social Expectation Rating (with interaction term) to exclude differential learning for the groups and to account for inter-individual influences of baseline ratings on expectation rating during acquisition. We calculated a Type-II-MANOVA using Pillai’s test statistic for the linear model. As expected, we found no significant interaction between Group and Baseline Social Expectation Rating, *F*(1,15) = 1.41, *p* = .264, and no significant main effect for Group, *F*(1,15) = 0.85, *p* = .593, but a main effect of the Baseline Social Expectation Rating, *F*(1,15) = 3.53, *p* = .013. Overall, the linear model accounted for 21% of variance (*R*^2^ = .21), which constitutes a medium effect (Ellis, 2010).

#### Hypothesis 2: Main Effect of Group in Stabilisation vs. Modification Phase

Following the significant interaction of Group x Stabilisation vs. Modification Phase in the main analyses, we constructed a linear model of the social expectation ratings (as outcome matrix for ratings 11 to 20) predicted by experimental condition to further investigate the main effect of Group. The Type-II-MANOVA revealed a marginally significant main effect for Group (*F*(1,17) = 2.38, *p* = .055). The model explained 19% of the variance (*R*^2^ = .19) constituting a medium effect (Ellis, 2010).

#### Exploratory Analysis: Stability of the Social Expectation Ratings

To test whether the social expectation ratings would remain consistent during test phase, we analysed a linear model of the social expectation ratings (outcome matrix for ratings 21 to 30) as a function of Group. As expected, the Type-II-MANOVA did not reveal a significant main effect for Group, *F*(1,17) = 0.88, *p* = .568. For test phase, the linear model accounted for 7% of the variance (*R*^2^ = .07) which constitutes a small effect (Ellis, 2010).

#### Suspiciousness of the Cover Story

Additionally, we analysed suspiciousness of the cover story. Seven participants reported doubts about the authenticity of the webcam conferences, six reported that they felt something ‘was off’ while 15 participants found nothing wrong with the webcam conferences. Further, three participants knew some of their ‘webcam partners’. However, a sensitivity analysis excluding all suspicious participants did not reveal significant differences in the result patterns. Therefore, we based our results on the whole sample.

## Discussion

While social rejection expectations play a crucial role in mental health, experimental research on the processes of how these expectations develop, maintain, and change is currently lacking. Our study addresses this gap by providing an ecologically valid and highly standardised experimental paradigm to investigate the acquisition, maintenance, and modification of situation-specific social rejection expectations in healthy samples. Results indicate, that this paradigm can be used to successfully induce (Hypothesis 1) as well as differentially change (Hypothesis 2) situation-specific social rejection expectations in healthy participants as a function of social feedback (social rejection vs. social appreciation). Altogether these results are consistent with the predictions drawn from the ‘ViolEx-Model’, which assumes modification of expectations after experiencing disconfirming results (e.g. positive social feedback after negative social feedback) as well as stabilisation of expectations after experiencing confirming results (e.g. Rief et al., 2015). Further, our results are in line with previous research on expectation development, maintenance, and modification in healthy participants. For example, Liebke et al. (2018) showed that healthy participants increase (respectively reduce) expectations of social acceptance as a function of social feedback (acceptance vs. rejection). Kube, Rief, Gollwitzer, and Glombiewski (2018) as well as Kube, Kirchner, Rief, Gärtner, and Glombiewski (2019) provided similar results concerning the modification of performance-related expectations as a function of performance-related feedback. Kube, Rief, Gollwitzer, and Glombiewski (2018) showed that healthy participants modify dysfunctional task-specific performance expectations in face of positive performance feedback. Consistently, Kube, Kirchner, Rief, Gärtner, and Glombiewski (2019) found that healthy as well as depressed participants update dysfunctional task-specific performance expectations in accordance to positive vs. negative feedback.

Moreover, our results resemble basic result patterns found in fear conditioning paradigms concerning the acquisition and modification of fear (Lissek et al., 2005): Repeatedly pairing self-presentation with social rejection led to higher social rejection expectation (i.e. higher ‘contingency awareness’, Lonsdorf et al., 2017, pp. 268-269) while social rejection expectations decreased in turn when social rejection feedback was omitted. However, comparability is limited here, since social expectations formed in the real world might interfere with social expectations formed within No1LikesU! (which is different from most typical fear conditioning procedures). Concerning our test phase, results indicate no ‘return’ or ‘renewal’ of social rejection expectations which is normally a common phenomenon in classical fear conditioning ('return of fear', Lonsdorf et al., 2017, p. 260). The stability of the associations learned within stabilisation vs. modification phase might be due to partial reinforcement during this phase as occasional reinforcement seem to attenuate return of fear in human fear conditioning (Craske et al., 2014; Culver, Stevens, Fanselow, & Craske, 2018).

### Limitations

Despite incorporating naturalistic stimuli, No1LikesU! does not provide dynamic social interactions. While the pre-scripted video stimuli ensure standardised experimental manipulation, these stimuli do not adapt to individual expressions of participants, threatening its external validity. Moreover, the paradigm only focuses on one specific social situation, i.e. self-disclosure in front of a stranger. Thus, investigating the generalisation of social rejection expectations to other social situations might be difficult within this paradigm. Additionally, a substantial amount of our participants seemed to be suspicious about the ‘webcam conferences’ and the social feedback we provided within No1LikesU!. While this issue could be solved at the expense of standardisation (for example by using real time interactions with confederates), problems with suspiciousness should not be overestimated within the actual procedure. Firstly, post-hoc questionnaires about the ‘aims and purposes’ of a study demand for suspiciousness by construction and therefore potentially overestimate actual suspiciousness of individuals during participation. Secondly, research on social exclusion shows that experiences of social exclusion stay impactful even if participants know that social feedback is simulated (e.g. Zadro, Williams, & Richardson, 2004). Further, we measured situation-specific social expectation only via self-report on a one-item scale. While expectations are usually assessed via self-report, more advanced self-report measures as well as multimodal indicators of social rejection expectation (e.g. avoidance behaviour) would improve validity of social rejection expectation assessment.

Further, while we incorporated general suggestions on fear conditioning paradigms, there are no clear instructions on how to set certain parameters in associative learning procedures (e.g. reinforcement rate or trial number). Thus, changing these parameters might also influence the effects of the paradigm.

Also, while we focused on contingency learning of outcome expectations, we did not include valence ratings for social rejection and social appreciation. Meta-analyses clearly show negative valence for social rejection (Gao et al., 2017), however, individual valence ratings might influence contingency learning. Outcome valence and outcome expectations might be coded differently in human brains (von Borries et al., 2013). While many brain areas associated with contingency learning seem independent of valence, some brain areas are suggested to be more strongly activated when processing positively evaluated stimuli (Bischoff-Grethe et al., 2009).

Finally, while we incorporated the concept of ‘expectation violation’ (Rief et al., 2015) in our paradigm, it could be argued that we did not provide real extinction training in our study as typically applied in fear conditioning (Lonsdorf et al., 2017). Since social rejection feedback was not only omitted but replaced by social appreciation feedback, we rather provided a ‘counterconditioning’ (de Jong, Vorage, & van den Hout, 2000) approach in group ‘Modification’.

### Future Directions

No1LikesU! provides options for broad applications to investigate the acquisition, maintenance, and modification of social rejection expectations within a highly standardised and ecologically valid experimental procedure. It is adaptable to various research attempts. Future research should use No1LikesU! to identify differences in the development, maintenance, and modification of social rejection expectations between healthy and clinical samples (with special regards to patients with borderline personality disorder, social anxiety or depression). To test whether clinical samples show to be differentially more sensitive to social rejection experiences during acquisition than healthy controls and show to be less responsive to social appreciation experiences during modification, has important implications for etiological considerations and clinical treatment. On the one hand, this could call for the development of expectation-focused etiological models (with special emphasise on dysfunctional social rejection expectations as connecting link) like Kube, Siebers, et al. (2018) as well as Rief and Joormann (2019) proposed for major depression. On the other hand, these results would stress the need for carefully designed expectation-focused psychological interventions specifically targeting dysfunctional social rejection expectations through contradictory experiences like Kube, Glombiewski, and Rief (2019) elaborated for people with depressive symptoms. Further, this would extend former findings on the ‘ViolEx-Model’ and clarify whether expectations of social rejection should be especially targeted in clinical practice. In order to develop proper interventions, researchers should apply No1LikesU! to investigate whether different interventions on informational processing (e.g. verbalisation, functional attention management) improve the modification of social rejection expectations in face of expectation violations. Here, it would also be of interest to investigate behavioural changes in participants (healthy participants as well as clinical samples) following social appreciation vs. social rejection feedback. This could provide further insight in behavioural expressions of social rejection expectations, which might also consolidate or even reinforce social rejection expectations.

From an ethical point of view, screening for and treatment of emotional distress produced by the paradigm should be enhanced when investigating clinical samples but also healthy controls. Researchers should provide extended debriefing and emotional aftercare by trained psychotherapists in order to prevent clinical subjects from transferring negative social experiences from the paradigm to their real life. Further, they should integrate phases of repeated positive social experiences at the end of their experiments by default in order to compensate for negative social experiences.

### Conclusion

No1LikesU! is an ecologically valid and highly standardised experimental paradigm to investigate the development, maintenance, and modification of social rejection expectations. Participants pass multiple short ‘webcam-conferences’ (video stimuli) in which they answer personal questions to different ‘listeners’ (confederates). Afterwards, they receive manipulated social feedback on their self-presentation. Our results suggest that researcher can use No1LikesU! to induce and alter social rejection expectations in healthy participants. Future research should focus on differences in the acquisition, maintenance, and modification of social rejection expectations between healthy and clinical samples. Additionally, incorporating interventions on expectation violation processing might improve the modification of social rejection expectations with implications for clinical treatment.

## Supplementary Materials

The supplementary material contains an overview of the 30 questions used in the No1LikesU! paradigm (Appendix A). Questions were adapted from various dating websites to promote positive self-disclosure. Appendix B provides the results of the Level-1- and Level-2-mixed effects models within the multilevel mixed effect multinomial linear regression (for access, see Index of Supplementary Materials below):

10.23668/psycharchives.3082 Supplement 1 Supplementary materials to "No1LikesU! – A pilot study on an ecologically valid and highly standardised experimental paradigm to investigate social rejection expectations and their modification"

D’AstolfoL.
KirchnerL.
RiefW.
 (2020). Supplementary materials to "No1LikesU! – A pilot study on an ecologically valid and highly standardised experimental paradigm to investigate social rejection expectations and their modification". PsychOpen. 10.23668/psycharchives.3082PMC964548936397828
