# Supplementary material for: No1LikesU! – A Pilot Study on an Ecologically Valid and Highly Standardised Experimental Paradigm to Investigate Social Rejection Expectations and Their Modification
Source: Clin Psychol Eur. 2020 Jun 30;2(2):e2997. doi: 10.32872/cpe.v2i2.2997 (PMC9645489; doi:10.32872/cpe.v2i2.2997)
Supplement: Supplement 1 [file cpe-02-2997-s1.pdf]

Supplementary Material

Appendix A: Personal Questions Used in the Paradigm

1) What are your hobbies?
2) How can one make you happy?
3) What would be your most favorite job and why?
4) Is there something you would love to try out in life?
5) How do you think your life will be in 20 years?
6) What do you like about other people's personalities?
7) Which person do you look up to and why?
8) What do you like to do in your spare time?
9) What do you like about yourself?
10) What kind of superpower would you like to have and why?
11) What is your favorite book and why do you like it so much?
12) What is your favorite movie and why do you like it so much?
13) If you could only take three personal items on a deserted island, which ones would you choose?
14) Imagine winning the lottery, what would you do with the money?
15) How does a perfect day look like for you?
16) How do perfect holidays look like for you?
17) Who would you like to switch life with for one day?
18) How would you describe yourself in three words and why?
19) What would be the perfect lifestyle for you
20) What's your passion?
21) What are you proud of?
22) Which three things does everybody need to try out in life?
23) What do you do to relax?
24) What does friendship mean to you?

25) Which skills or abilities did you always want to learn?

26) What do you need to feel comfortable?

27) If you could travel in time, which times would you be interested in and why?

28) If you opened a business, what would it be and why?

29) If you never had to sleep, what would you do with the extra time?

30) What are you probably better at than most people?

Appendix B: Results of the Level 1 and Level 2 Mixed Effects Models

The Level 1 linear regression revealed a significant effect for acquisition phase (β = -0.07, t = -4.96, $p < .001$), on social expectation rating. In other words, during the acquisition phase, expectation ratings decreased (indicating more social rejection expectations) with increasing webcam conferences. We did not find a significant main effect for the 'Modification' vs. 'Stabilization' phase (β = 0.01, t = 1.15, p = .24). Also, in accordance with our hypotheses, we did not find a significant main effect of test phase (β = 0.01, t = 1.26, p = .20), since we did not expect a change in expectation ratings when no further feedback occurred. We then analysed the table of variance of the Level 1 model against the null model. We found a significant effect for the acquisition phase ($F(1,864) = 23.23$, $p < .001$) and the 'Modification' vs. 'Stabilization' phase ($F(1,864) = 5.92$, $p = .01$). As expected we did not find a significant effect for the test phase ($F(1,846) = 1.58$, $p = .20$). When adding the random effect for participant (Level 2), we again found an effect for the acquisition phase (β = -0.07, t = -5.63, $p < .001$), indicating that social expectation ratings declined during the acquisition phase. Analogous to the Level 1 model, we did not find significant effects for the 'Modification' vs. 'Stabilization' phase (β = 0.01, t = 1.31, p = .19) and test phase (β = 0.01, t = 1.43, p = .15).
